# General population norms for the EQ-5D-3 L in Norway: comparison of postal and web surveys

**DOI:** 10.1186/s12955-018-1029-1

**Published:** 2018-10-19

**Authors:** Knut Stavem, Liv A Augestad, Ivar S Kristiansen, Kim Rand

**Affiliations:** 1 Health Services Research Unit, Akershus University Hospital, Lørenskog, Norway; 2 Department of Pulmonary Medicine, Akershus University Hospital, Lørenskog, Norway; 3 Campus Ahus, Institute of Clinical Medicine, Faculty of Medicine, University of Oslo, Lørenskog, Norway; 4 Department of Health Management and Health Economics, Faculty of Medicine, University of Oslo, Oslo, Norway

**Keywords:** EQ-5D, Health-related quality of life, Postal survey, Web survey, Population norms, General population, Utility, EQ-5D index, EQ VAS

## Abstract

**Background:**

The EQ-5D-3 L instrument is a standardized questionnaire which was developed as a simple, generic measure of health for clinical and economic appraisal. To aid in the interpretation, scores are often compared with a normative group. The objectives of this study were 1) to provide population norms for the EQ-5D-3 L for Norway, and 2) to compare scores from postal and web surveys.

**Methods:**

We conducted two surveys in samples that were aimed to be representative of the Norwegian general population: 1) a postal survey (*n* = 5000) and 2) a panel study with electronic data collection (*n* = 1936). For scoring the EQ-5D Index, we used the UK tariff. EQ-5D items were compared using multivariable ordinal logistic regression analysis and EQ-5D Index and EQ VAS scores using multivariable linear regression, adjusting for age, sex and education.

**Results:**

In total 1131 (22.6%) responded to the postal survey and 977 (50.5%) to the web survey. The odds ratio (OR) for being in a higher score category on the Pain/Discomfort scale in the web survey was 1.25 (95%CI 1.04 to 1.50, *p* = 0.019) relative to the postal survey. The odds were similar in the other four dimensions. The EQ-5D Index and EQ VAS scores were similar in the postal and web surveys in the various strata according to age, sex and education, except for lower unadjusted and adjusted score for web respondents aged 41–50 years and for those with higher education (≥14 years) than postal respondents.

**Conclusions:**

The distribution of scores for the EQ-5D descriptive system and its derived utility scores were rather similar in a postal survey and a panel web survey. Hence, these values were combined into a norm set for Norway.

## Background

Health-related quality of life (HRQOL) has become an increasingly important outcome of health care and public health interventions. HRQOL can be assessed using different instruments. The EQ-5D-3 L instrument is a standardized HRQOL questionnaire, which was developed by the EuroQol Group to provide a simple, generic measure of health for clinical and economic appraisal [1]. The EQ-5D-3 L questionnaire is available in more than 160 translated versions [2], and is the most commonly used instrument worldwide for assessment of utilities for use in cost-utility analyses to appraise health care interventions [3]. The EQ-5D is widely used in clinical trials, observational studies, and other health surveys [4, 5].

To aid in the interpretation of psychological tests, scores are often compared with a normative group, i.e. a group of test-takers that are representative of the population for whom the test is intended. Norms provide a frame of reference to interpret an individual’s scores relative to scores of others, whether the norms are national or local [6]. National population norms for the EQ-5D by socio-demographic characteristics are available from many countries [7], including the UK [8], Sweden [9], Finland [10], Denmark [11], Italy [12], Poland [13], Switzerland [14], United States [15, 16], China [17], Japan [18], Sri Lanka [19] and more [7]. The population norms present distributions of the five item scores, EQ VAS scores, or EQ-5D Index values that are based on different value sets and methods, e.g. the European VAS value set algorithms, or country-specific VAS or time trade-off (TTO)-derived values [7].

Most national and regional EQ-5D population surveys have used face-to-face interviews or computer-assisted personal interviews [7]; however some studies used postal surveys [20–23] or computer-assisted telephone interviews [12, 24, 25]. Some studies also pool data from different postal surveys, for example to establish population norms for the EQ-5D [11]. For another commonly used health status measure, the SF-36 version 1, data from telephone and face-to-face interviews were combined to represent US general population norms [26]. However, we are not aware of population norms from web-based surveys for the EQ-5D.

Several Norwegian studies have used the EQ-5D-3 L to examine HRQOL in many diseased populations, including people with epilepsy [27], COPD [28], stroke [29, 30], diabetes [31], rheumatoid arthritis [32], hip fractures [33–35], HIV/AIDS [36], brain tumors [37], and multiple myeloma [38]. To date there are no available population norms for this instrument in Norway.

This study aimed to provide population norms for the EQ-5D-3 L according to age, and sex, using nationally representative samples of Norwegians aged 18 years and above. The study compared results from a postal survey and a web-based data collection. We hypothesized that there would be no difference between the postal and web-based surveys after adjustment for age, sex and education.

## Methods

### Samples and surveys

The study consisted of two study arms, 1) a postal survey and 2) a panel study with electronic data collection. We sent invitations in both surveys to samples that were aimed to be reasonably representative of the Norwegian general population with regard to sex, age and education (details below). The postal survey was carried out from May 10 to June 18, 2010, and the electronic survey from June 7 to June 22, 2010. In both surveys, we sent one reminder.

The authors prepared a common core questionnaire that was adapted for postal and electronic data collection by TNS Gallup, a market research company. The postal and panel versions of the questionnaire were similar, with the exception of how the respondents responded to the EQ VAS, a valuation of health conditions on a 0–100 point scale. The postal version had a vertical visual analog scale, while the electronic version used a solution with a horizontal slider. When moving the slider from left to right, the respondent decided on a value between 0 and 100.

The study was approved by the local privacy ombudsman for research at Akershus University Hospital on 27 April 2010, noting that the project was considered to be anonymous information, and presentation for the Regional Committee for Medical Health Research Ethics was not considered necessary.

#### Postal survey

The list of names and associated registered addresses was selected from the National Registry of the Norwegian Tax Administration (Folkeregisteret). A company, EDB Businesspartner, was after application granted access to the registry and selected a random sample of 5000 persons from the total Norwegian population aged 19–100 including age, postal address, and marital status. TNS Gallup was responsible for data collection, including carrying out the postal survey using the name/address list. After the survey the responses were anonymized before the data file was given to the authors. Hence, we only had access to age, sex, marital status and county of habitation from the registry. The postal questionnaire had an enclosed return envelope. Half of the participants in addition received a lottery ticket with a value of NOK 25 (≈ 2.5 Euros) as an incentive to respond.

#### Panel survey with electronic data collection

The sample for the electronic data collection was drawn from TNS Gallup’s access panel, “GallupPanelet”. This panel included about 60,000 willing survey participants. They had been recruited through various previous telephone (landline and mobile phone) and postal surveys conducted by TNS Gallup. The panel was maintained continuously by recruitment, updating of background variables and automatic withdrawal of participants that had completed a certain number of surveys. Because of the size and recruitment method it was possible to select nationally representative samples > 18 years of age. Such samples were selected in two steps; first strata according to age, gender, address were created; then the final sample was selected within the strata.

The response rate in a panel survey cannot easily be compared with the response rate in a postal survey. The panel participants have been recruited in advance, and the survey is closed when the required number of respondents has been achieved.

The electronic part of the data collection used an electronic HTML-based questionnaire. We invited potential participants through an e-mail invitation, with a link to the questionnaire. The respondents to the panel were compensated by an incentive, where they received points according to a normed length of the questionnaire in minutes (1 point = 1 min). The points could be collected as a gift card or donated to a charity. The value of 1 point was NOK 1 (0.1 Euro) at the time of the survey.

### The EQ-5D instrument

The EQ-5D instrument is a self-completed questionnaire, which can be completed on paper, tablet or on the web. It consists of the EQ-5D descriptive system that measures HRQOL today on five dimensions (mobility, self-care, usual activities, pain/discomfort, and anxiety/depression). This study used the 3 L version, EQ-5D-3 L, having three scoring levels in each dimension. In addition, the instrument contains a 20 cm vertical visual analog scale, the EQ VAS, rating “your own health state today” on a 0 (worst imaginable) to 100 (best imaginable) scale.

Based on the responses to the descriptive system, the scores can be aggregated to a single index value, the EQ-5D Index, which can be used in the estimations of Quality-Adjusted Life Years (QALYs). The EQ-5D Index is generated on the basis of an algorithm reflecting the preferences of general populations, or tariffs. Several country-specific tariffs exist. As no tariff is available for Norwegians, this study used the oldest and most commonly used tariff, the UK tariff [39], for generating EQ-5D Index values in the Norwegian population.

### Statistical analysis

We compared descriptive statistics for the respondents in the postal and web surveys using the *t*-test or Fisher’s exact test and the distributions of scores on the EQ-5D single items using the Fisher’s exact test.

To adjust for age, sex, and education, we attempted to use ordered logistic regression analysis with the ordinal EQ-5D dimension scores as the dependent variable. Because few respondents scored at level 3 in the Mobility and Self-care dimensions, we combined the “Some problems/Confined to bed” for the Mobility dimension, and the “Some problems/Unable to wash or dress” for the Self-care dimension. For these two dimensions, the survey results were compared using multivariable logistic regression analysis. In the three remaining dimensions, Usual activities, Pain/Discomfort, and Anxiety/Depression, we used ordered logistic regression analysis. The proportional odds assumption was checked using the Brant test and was met. In these models, we used three co-variates (age (continuous), sex and education (< 10 years, 10–13 years, > 13 years).

We compared EQ VAS, EQ-5D Index scores and EQ VAS-based values using the *t*-test for unadjusted scores, and used multivariable linear regression analysis to adjust for age, sex and education.

We used Stata version 14.2 (StataCorp, College Station, TX) or R version 3.2.0 (The R Foundation for Statistical Computing) for statistical analyses. A significance level of 5% was chosen, using two-sided tests.

## Results

### Response and representativeness

In the postal survey, 101 questionnaires among 5000 were returned because of unknown address. In total 1276 individuals responded to the questionnaire, of whom 1131 (22.6% of the gross sample, 23.1% of the net sample) completed both the EQ-5D and the EQ-VAS and were used in the further analysis.

The panel participants were recruited in advance, and the survey was closed when the targeted number of respondents had been reached. Of a total of 1936 panel participants that received the invitation, 1192 opened the survey, and 1003 (51.8%) completed it, and 977 had valid responses to the EQ-5D items. The response rate in the electronic survey cannot easily be compared with the response rate in a postal survey.

The final samples had a good spread according to sex, age and educational attainment. The respondents had a mean (SD) age of 51.8 (17.4) and 50.7 (14.7) years in the postal (*n* = 1131) and web (*n* = 977) survey, respectively. Age ranged from 19 to 97 years in the postal and 19 to 86 years in the web survey. The age distribution between the respondents in the two surveys differed (*p* < 0.001), with more respondents in the 61–70 years group (26%) and fewer > 70 years (6%) in the web survey than in the postal one (18% and 15%, respectively). Also the distribution of educational attainment differed (*p* < 0.001), as the web survey had a larger proportion of respondents with basic education and lower proportion with higher education (Table 1). The proportion of female respondents was similar in both surveys (*p* = 0.56).

**Table 1:** Descriptive statistics for respondents. Number (%) unless stated otherwise

	Postal survey						Web survey						Pooled					
	Male		Female		Total		Male		Female		Total		Male		Female		Total	
*n*	*556*	*(49)*	*575*	*(51)*	*1131*	*(100)*	*468*	*(48)*	*509*	*(52)*	*977*	*(100)*	*1024*	*(49)*	*1084*	*(51)*	*2108*	
Age, years																		
19–30	61	(11)	86	(15)	147	(13)	43	(9)	65	(13)	108	(11)	104	(10)	151	(14)	255	(12)
31–40	73	(13)	96	(17)	169	(15)	71	(15)	74	(15)	145	(15)	144	(14)	170	(16)	314	(15)
41–50	110	(20)	103	(18)	213	(19)	100	(21)	99	(19)	199	(20)	210	(21)	202	(19)	412	(20)
51–60	106	(19)	121	(21)	227	(20)	114	(24)	102	(20)	216	(22)	220	(21)	223	(21)	443	(21)
61–70	110	(20)	90	(16)	200	(18)	114	(24)	138	(27)	252	(26)	224	(22)	228	(21)	452	(21)
≥ 71	96	(17)	79	(14)	175	(15)	26	(6)	31	(6)	57	(6)	122	(12)	110	(10)	232	(11)
Education, years																		
Basic education (≤10)	60	(11)	61	(11)	121	(11)	145	(31)	174	(34)	319	(33)	205	(20)	235	(22)	440	(21)
Secondary education (11–13)	262	(47)	231	(40)	493	(44)	211	(45)	198	(39)	409	(42)	473	(46)	429	(40)	902	(43)
Higher education (≥14)	216	(39)	260	(45)	476	(42)	112	(24)	137	(27)	249	(25)	328	(32)	397	(37)	725	(34)

### Comparison of scores between surveys

The distribution of scores in the Mobility, Self-care, Usual activities, and Anxiety/Depression dimensions did not differ between the two surveys, both for unadjusted scores and in the odds of being in a higher score category after adjustment for age, sex and education (Table 2). There was, however, a difference in the scores in the Pain/Discomfort dimension, with a larger proportion reporting no pain (level 1) in the postal survey than in the electronic one.

**Table 2:** Distribution of item scores according to sample, number (%), and comparison of scores between modes of survey

	Postal survey		Web survey		*P* ^a^	Odds ratio	95% CI	*P*
*n*	*1131*		*977*					
Mobility					0.66	0.97^b^	(0.74 to 1.28)	0.84
No problems	968	(86)	831	(85)				
Some problems	163	(14)	145	(15)				
Confined to bed	–		1	(0)				
Self-care					0.94	0.81^b^	(0.44 to 1.48)	0.49
No problems	1102	(97)	954	(98)				
Some problems	28	(3)	23	(2)				
Unable to wash or dress	1	(0)	–					
Usual activities					0.14	1.08^c^	(0.85 to 1.38)	0.52
No problems	949	(84)	789	(81)				
Some problems	172	(15)	175	(18)				
Unable to perform	10	(1)	13	(1)				
Pain/Discomfort					0.002	1.25^c^	(1.04 to 1.50)	0.019
No pain or discomfort	647	(57)	495	(51)				
Moderate pain or discomfort	444	(39)	426	(44)				
Extreme pain or discomfort	40	(4)	56	(6)				
Anxiety/Depression					0.62	0.88^c^	(0.70 to 1.10)	0.26
Not anxious or depressed	893	(79)	766	(78)				
Moderately anxious or depressed	228	(20)	198	(20)				
Extremely anxious or depressed	10	(1)	13	(1)				

^a^Fisher’s exact test; ^b^Logistic regression: OR for web compared to postal of scoring Moderate/extreme vs. Mild, adjusted for age, sex and education;

^c^Ordinal logistic regression: OR for web compared to postal of scoring in a higher category on the scale, adjusted for age, sex and education

EQ-VAS scores were similar in the postal and web surveys in the various strata according to age, sex and education, except for lower unadjusted and adjusted score for web respondents aged 41–50 years and for those with higher education (≥14 years) than for postal respondents (Table 3).

**Table 3:** EQ-VAS scores (0–100 scale) according to sample in different strata and comparisons of scores between modes of survey using linear regression analysis

	*n*	Postal survey		*n*	Web survey		*P*	
		Mean	SD		Mean	SD	Bivariate	Multivariable
Overall	*1131*	79.5	(18.8)	*977*	77.9	(19.5)	0.061	0.487^A,S,E^
Sex								
Male	556	79.3	(18.1)	468	77.6	(19.1)	0.152	0.626^A,E^
Female	575	79.7	(19.5)	509	78.2	(20.0)	0.217	0.679^A,E^
Age group, years								
19–30	147	86.1	(12.0)	108	83.6	(12.4)	0.105	0.106^S,E^
31–40	169	83.3	(15.9)	145	81.2	(17.7)	0.270	0.709^S,E^
41–50	213	83.1	(14.7)	199	77.8	(20.4)	0.003	0.011^S,E^
51–60	227	80.1	(17.3)	216	76.1	(21.0)	0.028	0.516^S,E^
61–70	200	76.9	(19.1)	252	76.3	(20.0)	0.713	0.476^S,E^
≥ 71	175	68.0	(25.6)	57	73.5	(21.3)	0.146	0.149^S,E^
Education, years								
Basic education (≤10)	121	69.1	(23.4)	319	74.6	(22.8)	0.023	0.360^A,S^
Secondary education (11–13)	493	77.9	(19.1)	409	78.3	(17.7)	0.747	0.740^A,S^
Higher education (≥14)	476	84.5	(13.7)	249	81.4	(17.1)	0.009	0.006^A,S^

^A,S,E^Controlling for Age (A), Sex (S), and Education (E) in multivariable linear regression

Comparison of UK EQ-5D tariff scores between the surveys showed a similar pattern, with lower unadjusted and adjusted scores in the web survey for respondents aged 41–50 years and those with higher education (≥14 years) than for postal respondents (Table 4).

**Table 4:** EQ-5D index scores using the UK tariff according to sample in different strata, and comparison between modes of survey using linear regression analysis

	*n*	Postal survey		*n*	Web survey		*P*	
		Mean	SD		Mean	SD	Bivariate	Multivariable
Overall	*1131*	0.848	(0.200)	*977*	0.821	(0.226)	0.003	0.288^A,S,E^
Sex								
Male	556	0.862	(0.191)	468	0.838	(0.218)	0.069	0.584^A,E^
Female	575	0.835	(0.208)	509	0.804	(0.233)	0.025	0.408^A,E^
Age group, years								
19–30	147	0.910	(0.135)	108	0.899	(0.148)	0.527	0.962^S,E^
31–40	169	0.873	(0.219)	145	0.866	(0.201)	0.778	0.421^S,E^
41–50	213	0.877	(0.162)	199	0.813	(0.233)	0.001	0.019^S,E^
51–60	227	0.830	(0.227)	216	0.775	(0.263)	0.019	0.131^S,E^
61–70	200	0.825	(0.200)	252	0.813	(0.220)	0.528	0.767^S,E^
≥ 71	175	0.785	(0.209)	57	0.788	(0.214)	0.911	0.693^S,E^
Education, years								
Basic education (≤ 10)	121	0.750	(0.240)	319	0.773	(0.267)	0.415	0.925^A,S^
Secondary education (11–13)	493	0.834	(0.206)	409	0.829	(0.207)	0.715	0.652^A,S^
Higher education (≥ 14)	476	0.895	(0.161)	249	0.868	(0.184)	0.041	0.028^A,S^

^A,S,E^Controlling for Age (A), Sex (S), and Education (E) in multivariable linear regression

### Normative scores

The distributions of the pooled EQ-5D item scores from the two surveys are presented as norm sets according to age and sex, indicating a lower proportion level 1 (lowest) score with increasing age, except for in the Anxiety/Depression dimension (Table 5). Normative scores are shown separately for men (Table 6) and women (Table 7).

**Table 5:** Distribution of EQ-5D item scores. Men and women according to age. Pooled postal and web surveys. Number (%)

	Age group, years													
	19–30		31–40		41–50		51–60		61–70		≥ 71		All	
*n*	*255*	(100)	*314*	(100)	*412*	(100)	*443*	(100)	*452*	(100)	*232*	(100)	*2108*	(100)
Mobility														
No problems	244	(96)	289	(92)	360	(87)	383	(87)	372	(82)	151	(65)	1799	(85)
Some problems	11	(4)	24	(8)	52	(13)	60	(14)	80	(18)	81	(35)	308	(15)
Confined to bed	0	(0)	1	(0)	0	(0)	0	(0)	0	(0)	0	(0)	1	(0)
Self-care														
No problems	254	(100)	308	(98)	405	(98)	430	(97)	441	(98)	218	(94)	2056	(98)
Some problems	1	(0)	6	(2)	7	(2)	13	(3)	11	(2)	13	(6)	51	(2)
Unable to wash or dress	0	(0)	0	(0)	0	(0)	0	(0)	0	(0)	1	(0.4)	1	(0)
Usual activities														
No problems	234	(92)	273	(87)	339	(82)	360	(81)	365	(81)	167	(72)	1738	(82)
Some problems	19	(8)	40	(13)	68	(17)	76	(17)	84	(19)	60	(26)	347	(17)
Unable to perform	2	(1)	1	(0)	5	(1)	7	(2)	3	(1)	5	(2)	23	(1)
Pain/Discomfort														
No pain or discomfort	196	(77)	214	(68)	227	(55)	209	(47)	205	(45)	91	(39)	1142	(54)
Moderate pain or discomfort	58	(23)	89	(28)	171	(42)	198	(45)	223	(49)	131	(57)	870	(41)
Extreme pain or discomfort	1	(0)	11	(4)	14	(3)	36	(8)	24	(5)	10	(4)	96	(5)
Anxiety/Depression														
Not anxious or depressed	190	(75)	233	(74)	321	(78)	351	(79)	377	(83)	187	(81)	1659	(79)
Moderately anxious or depressed	62	(24)	75	(24)	87	(21)	85	(19)	72	(16)	45	(19)	426	(20)
Extremely anxious or depressed	3	(1)	6	(2)	4	(1)	7	(2)	3	(1)	0	(0)	23	(1)

**Table 6:** Distribution of EQ-5D item scores. Men only, according to age. Pooled postal and web surveys. Number (%)

	Age group, years													
	19–30		31–40		41–50		51–60		61–70		≥ 71		All	
*n*	*104*	(100)	*144*	(100)	*210*	(100)	*220*	(100)	*224*	(100)	*122*	(100)	*1024*	(100)
Mobility														
No problems	102	(98)	137	(95)	189	(90)	188	(86)	190	(85)	78	(64)	884	(86)
Some problems	2	(2)	7	(5)	21	(10)	32	(15)	34	(15)	44	(36)	140	(14)
Confined to bed	0	(0)	0	(0)	0	(0)	0	(0)	0	(0)	0	(0)	0	(0)
Self-care														
No problems	104	(100)	142	(99)	209	(100)	211	(96)	220	(98)	114	(93)	1000	(98)
Some problems	0	(0)	2	(1)	1	(0.5)	9	(4)	4	(2)	8	(7)	24	(2)
Unable to wash or dress	0	(0)	0	(0)	0	(0)	0	(0)	0	(0)	0	(0)	0	(0)
Usual activities														
No problems	102	(98)	133	(92)	182	(87)	180	(82)	187	(84)	90	(74)	874	(85)
Some problems	2	(2)	11	(8)	27	(13)	34	(16)	35	(16)	28	(23)	137	(13)
Unable to perform	0	(0)	0	(0)	1	(0.5)	6	(3)	2	(1)	4	(3)	13	(1)
Pain/Discomfort														
No pain or discomfort	85	(82)	106	(74)	129	(61)	105	(48)	115	(51)	54	(44)	594	(58)
Moderate pain or discomfort	19	(18)	35	(24)	79	(38)	93	(42)	101	(45)	63	(52)	390	(38)
Extreme pain or discomfort	0	(0)	3	(2)	2	(1)	22	(10)	8	(4)	5	(4)	40	(4)
Anxiety/Depression														
Not anxious or depressed	85	(82)	116	(81)	167	(80)	175	(80)	193	(86)	102	(84)	838	(82)
Moderately anxious or depressed	19	(18)	27	(19)	40	(19)	41	(19)	30	(13)	20	(16)	177	(17)
Extremely anxious or depressed	0	(0)	1	(1)	3	(1)	4	(2)	1	(0)	0	(0)	9	(1)

**Table 7:** Distribution of EQ-5D item scores. Women only, according to age. Pooled postal and web surveys. Number (%)

	Age group, years													
	19–30		31–40		41–50		51–60		61–70		≥ 71		All	
*n*	*151*	(100)	*170*	(100)	*202*	(100)	*223*	(100)	*228*	(100)	*110*	(100)	*1084*	(100)
Mobility														
No problems	142	(94)	152	(89)	171	(85)	195	(87)	182	(80)	73	(66)	915	(84)
Some problems	9	(6)	17	(10)	31	(15)	28	(13)	46	(20)	37	(34)	168	(16)
Confined to bed	0	(0)	1	(1)	0	(0)	0	(0)	0	(0)	0	(0)	1	(0)
Self-care														
No problems	150	(99)	166	(98)	196	(97)	219	(98)	221	(97)	104	(95)	1056	(97)
Some problems	1	(1)	4	(2)	6	(3)	4	(2)	7	(3)	5	(5)	27	(3)
Unable to wash or dress	0	(0)	0	(0)	0	(0)	0	(0)	0	(0)	1	(1)	1	(0)
Usual activities														
No problems	132	(87)	140	(82)	157	(78)	180	(81)	178	(78)	77	(70)	864	(80)
Some problems	17	(11)	29	(17)	41	(20)	42	(19)	49	(22)	32	(29)	210	(19)
Unable to perform	2	(1)	1	(1)	4	(2)	1	(0)	1	(0)	1	(1)	10	(1)
Pain/Discomfort														
No pain or discomfort	111	(74)	108	(64)	98	(49)	104	(47)	90	(40)	37	(34)	548	(51)
Moderate pain or discomfort	39	(26)	54	(32)	92	(46)	105	(47)	122	(54)	68	(62)	480	(44)
Extreme pain or discomfort	1	(1)	8	(5)	12	(6)	14	(6)	16	(7)	5	(5)	56	(5)
Anxiety/Depression														
Not anxious or depressed	105	(70)	117	(69)	154	(76)	176	(79)	184	(81)	85	(77)	821	(76)
Moderately anxious or depressed	43	(29)	48	(28)	47	(23)	44	(20)	42	(18)	25	(23)	249	(23)
Extremely anxious or depressed	3	(2)	5	(3)	1	(1)	3	(1)	2	(1)	0	(0)	14	(1)

The UK EQ-5D tariff scores for Norway are shown separately for the postal, web and pooled surveys (Table 8), indicating a consistent pattern across age and gender, with two exceptions: 1) a slight dip in scores for males aged 51–60 years in both surveys, when compared with similarly aged women and with younger and older males, and 2) a dip in scores for females aged 41–50 in the web survey.

**Table 8:** UK EQ-5D index scores for Norway, by panel, sex, and age. Mean (SD)

	Postal survey (*n = 1131*)						Web survey (*n = 977*)						Pooled (postal + web surveys) (*n = 2108*)					
	Male		Female		All		Male		Female		All		Male		Female		All	
Age group, years																		
19–30	0.928	(0.104)	0.898	(0.152)	0.910	(0.135)	0.951	(0.088)	0.865	(0.169)	0.899	(0.148)	0.938	(0.098)	0.884	(0.160)	0.906	(0.140)
31–40	0.920	(0.141)	0.837	(0.259)	0.873	(0.219)	0.885	(0.183)	0.848	(0.217)	0.866	(0.201)	0.903	(0.164)	0.842	(0.241)	0.870	(0.211)
41–50	0.897	(0.140)	0.855	(0.181)	0.877	(0.162)	0.855	(0.189)	0.771	(0.263)	0.813	(0.233)	0.877	(0.166)	0.814	(0.229)	0.846	(0.202)
51–60	0.818	(0.253)	0.841	(0.202)	0.830	(0.227)	0.767	(0.282)	0.785	(0.242)	0.775	(0.263)	0.791	(0.269)	0.816	(0.223)	0.804	(0.247)
61–70	0.848	(0.180)	0.798	(0.220)	0.825	(0.200)	0.837	(0.197)	0.792	(0.235)	0.813	(0.220)	0.843	(0.189)	0.794	(0.229)	0.818	(0.211)
≥ 71	0.798	(0.217)	0.769	(0.200)	0.785	(0.209)	0.778	(0.215)	0.797	(0.216)	0.788	(0.214)	0.794	(0.215)	0.776	(0.204)	0.786	(0.210)

## Discussion

This is the first study to examine the Norwegian population norms for the EQ-5D instrument. The major finding was that the distribution of scores for the EQ-5D descriptive system and its derived utility scores were rather similar in a postal survey and a panel survey with electronic data collection. Therefore, we think it is justified to pool the values in a combined norm set.

In the two surveys, and hence in the pooled sample, there was a deterioration of health status with increasing age, with some minor exceptions, as shown in previous studies and in a catalogue of population norms for the EQ-5D index and the EQ-VAS [7]. We noted a dip in EQ-5D index scores in the 51–60 years group among males, or an increase in the 61–70 years group. Another study noted a similar dip in EQ-5D index and other HRQoL scores at ages 55–64, or a slight hump in HRQoL in the 65–74 age group, compared to a linear, downward trend associated with age group; however, this was not considered a systematic effect [16]. The difference between genders in the present study was consistent across the EQ-5D index and EQ-VAS, in line with previous studies [7].

As far as we know, no previous collection of EQ-5D-3 L population norms has been collected using a web sample. Therefore, the difference in results between web-based and postal administrations is unknown. In principle, using a web panel could be problematic for two reasons: First, the mode of administration could impact on how people respond. Second, a web panel is by definition self-selected, and the question is whether the sample is representative of the general population.

Addressing the first question, a review of papers studying the agreement between electronic and paper administration of patient-reported outcome measures, including EQ-5D-3 L, found that the two modes of administration had a high level of equivalence [40]. The review was mostly based on studies of diseased populations, however, there is no obvious reason why the general population should have a larger difference in responses based on mode of administration than patients. Increasingly, patients who participate in studies fill out the EQ-5D electronically, and the new standard for performing valuation studies involves the use of computer-assisted interviews [41]. In that respect, electronically collected population norms have ecological validity since the mode of administration is the same.

McHorney et al. noted that in a randomized trial on the effects of data collection method on the summary scores of a different health status instrument, the SF-36, respondents to telephone interviews tended to score higher on the MCS than those in a postal survey, although there was no effect on the PCS [42]. Furthermore, telephone-administered US general population norms for the SF-36v2 in 2005–2006 [43] were higher than for norms collected by mail administration in 1998 [44] which was attributed to the effect of telephone administration.

Response rates to epidemiological surveys in general [45] and in the Nordic countries have declined over the last decades [46]. The consequences of the use of web surveys have been debated and challenges raised [47]. Often, results obtained with electronic versions are generally similar to paper versions, in terms of outcomes and psychometric properties, although the response rates to internet surveys tend to be lower and the respondents less representative than in mail surveys [48, 49]. However, established offline norms for established offline tests may still not be appropriate for use with online versions, as evidenced from psychometric tests in different settings [50]. Moreover, Buchanan states that when comparing online scores with normative data, “normative data gathered online must be used” [50]. This may apply for HRQoL as well.

Self-selection bias is a general problem when conducting surveys; people who agree to participate in surveys are systematically different from people who decline [51]. However, one could assume that web panels consist of individuals who are the most inclined to participate in studies. To counter this effect, survey companies are able to put extra effort into recruiting individuals with typical non-responder characteristics to their panels, for instance individuals from the youngest and oldest age groups. As an additional step in studies where representativeness is important, the companies may over-sample groups which have low response rates.

The pooled postal and web sample in this study was comparable to the Norwegian general population regarding gender and education, although the proportion aged 19–40 years was somewhat lower and the proportions 41–60 and > 60 years of age were somewhat higher, than in the general population in 2010 [52]. We did not weight the sample to better approximate the general population. Some studies of HRQoL have pointed at various method for adjusting web-based panel results to better approximate the scores of the general population, such as post-stratification adjustment [53–55]. One of these studies reported web-based norms for two HRQoL instruments, the AQoL-6D and AQoL-8D, using post-stratification weights to address the effects of self-selection in the web-based survey [55].

Internet coverage is important when assessing the potential bias in using a web panel, since in some cases only selected parts of a population – the wealthy and highly educated, has had access to the relevant technology. In Norway, using computers connected to the internet is free in public libraries. Further, there is a high coverage rate of internet in Norway, as in 2010 90% of the Norwegian households had access to the internet, and 97% in 2016 [56].

Recently, the Norwegian Medicines Agency has published draft guidelines for pharmaco-economic analyses [57]. The agency recommends using the EQ-5D for estimating utilities, and suggests using Swedish population norms [9, 23] as a reference, because Norwegian norms are unavailable. The means and SDs for the population values in the various age strata in the present study were very similar to the Swedish norms collected in 1998 [9]. The norms in both the Swedish and the present study were based on the same EQ-5D-3 L tariff from the UK [39], although the Swedish study assigned a value of 0 to states rated as worse than death [9], in contrast to the present study and the original UK tariff. There are also other important methodological differences. For example, the Swedish norms were based on regional data only (from Stockholm County) from a postal survey, and it had three postal reminders and a telephone follow-up of non-respondents resulting in a higher response rate than in the present study. Furthermore, the Swedish study presented data separately for those > 80 years of age [9].

Some limitations of this study should be noted. We have discussed some of the short-comings and problems with a web-based panel, where it is difficult to evaluate the non-response and representativeness as traditionally done in postal surveys. The sample size was sufficient to provide normative values according to sex and age groups in 10-year intervals, although the number of respondents above > 70 years was too small for splitting this into several age groups. This may be important, as health status might be expected to deteriorate rapidly with increasing age > 70 years.

## Conclusion

Our observations of equivalence between general population normative values collected in a web panel aimed to be representative of the general population and those from a postal survey, in which the survey was sent to random individuals drawn from the Population Register of Statistics Norway, supports the use of web samples when collecting EQ-5D population norms. Because of the similarity of the scores, we have pooled the scores to a larger data set, which may be used as general population norms for the EQ-5D-3 L in Norway. This may be useful for example in assessment of health care interventions or in pharmaco-economic analyses.

## Abbreviations

HRQoL: Health-related quality of life
NOK: Norwegian kroner
OR: Odds ratio
SD: Standard deviation
VAS: Visual analog scale
